# Assessment of left ventricular volume overload: comparison of 2-D echo and cardiac MRI

**DOI:** 10.1186/1532-429X-16-S1-P245

**Published:** 2014-01-16

**Authors:** Julianne Matthews, Nicholas C Boniface, Brandon M Mikolich, John Lisko, J Ronald Mikolich

**Affiliations:** 1 Northeast Ohio Medical University, Rootstown, Ohio, USA; 2 Sharon Regional Health System, Sharon, Pennsylvania, USA

## Background

Left ventricular cavity dimensions and volumes are useful parameters to diagnose and monitor cardiac pathologies which lead to LV volume overload. 2-D echo/Doppler is the most commonly used imaging modality to assess LV volume overload in patients with valvular regurgitant disease. However, 2-D echo suffers from inherent limitations related to adequacy of the intercostal echo window, anatomic positioning for cavity dimensions and volumes and incomplete visualization of the entire left ventricular cavity. This study was designed to compare assessment of LV cavity dimensions and volumes by 2-D echo and cardiac magnetic resonance (CMR) techniques in patients with LV volume overload due to valvular regurgitation

## Methods

An institutional cardiac imaging database was queried to identify patients with at least moderate (2+ or greater) mitral regurgitation or aortic regurgitation who had 2-D and CMR studies within 6 months of each other. LV end-diastolic(LVIDd) and end-systolic(LVIDs) dimensions were tabulated, along with LV end-diastolic(LVEDV) and end-systolic(LVESV) volumes and severity of valvular regurgitation (1+ to 4+ scale) for both 2-D and CMR. 2-D regurgitation was estimated by Doppler analysis and CMR regurgitation was estimated by calculation of the regurgitant fraction using phase velocity mapping. The mean values for LVIDd, LVIDs, LVEDV and LVESV by 2-D and CMR were computed and statistically compared using a paired-sample t-test. Each parameter was also analyzed as a function of severity of regurgitation.

## Results

**Figure 1:**
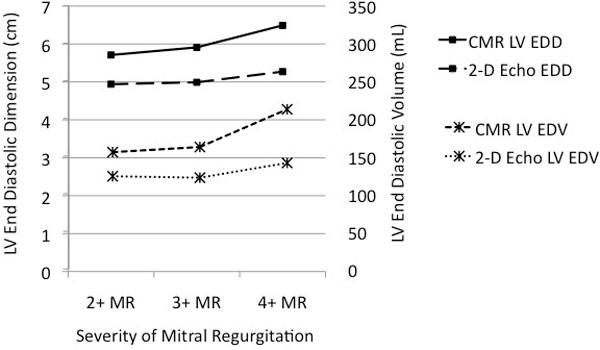
**Relationship of LV End-Diastolic Dimension and Volume to Severity of Mitral Regurgitation By 2-D Echo and CMR Methods**.

Of 1,817 patients in the CMR database, 102 patients met study inclusion criteria, 52 with AR and 63 with MR. The mean LVIDd by CMR was 5.87 cm and 5.09 cm by 2-D (p < 0.001). The mean LVIDs by CMR was 4.03 cm and 3.46 cm by 2-D (p < 0.001). The mean LVEDV by CMR was 166.16 ml and 129.48 ml by 2-D (p < 0.001). The mean LVESV by CMR was 77.71 cm and 56.06 ml by 2-D (p < 0.001). Parameter analysis as a function of severity of regurgitation is shown in the Figure 1.

## Conclusions

In patients with LV volume overload due to valvular regurgitation, 2-D echo significantly underestimates LVIDd, LVIDs, LVEDV and LVESV, resulting in over-estimation of global LV ejection fraction. In patients with mitral regurgitation, increases in LV end-diastolic dimension and LV end-diastolic volume on CMR are indicative of worsening regurgitation. A similar relationship to severity of mitral regurgitation could not be demonstrated for these parameters when assessed by 2-D echo techniques.

## Funding

None.

